# Correlation between comfort and medication adherence in people with type 2 Diabetes Mellitus: a cross-sectional study

**DOI:** 10.1590/0034-7167-2024-0449

**Published:** 2026-07-17

**Authors:** Rafael Oliveira Pitta Lopes, Simone Paulino Ferreira Coimbra, Hudson Carmo de Oliveira, Juliana Faria Campos, Renan Alves Silva, Marcos Antônio Gomes Brandão

**Affiliations:** I Universidade Federal do Rio de Janeiro. Rio de Janeiro, Rio de Janeiro, Brazil; II Universidade Federal do Rio de Janeiro. Macaé, Rio de Janeiro, Brazil; III Universidade Federal de Campina Grande. Cajazeiras, Paraíba, Brazil

**Keywords:** Medication Adherence, Patient Comfort, Type 2 Diabetes *Mellitus*, Nursing Theory, Insulin., Adherencia a la Medicación, Comodidad del Paciente, Diabetes *Mellitus* Tipo 2, Teoría de Enfermería, Insulina.

## Abstract

**Objectives:**

to test a hypothetical correlation between the comfort construct of Kolcaba’s Comfort Theory and the adherence construct to the use of oral antidiabetic drugs and insulin in people with type 2 Diabetes *Mellitus*.

**Methods:**

an analytical cross-sectional study was conducted with 198 participants. Comfort and medication adherence were measured, respectively, using the General Comfort Questionnaire, the Insulin Treatment Adherence Measure, and the Oral Antidiabetic Treatment Adherence Measure. Data were analyzed using descriptive and inferential statistics. Pearson’s correlation test was used for correlation analysis.

**Results:**

the mean medication adherence score was 5.22, and the mean comfort score was 130.25 among those using exclusively oral antidiabetic drugs, while those using insulin had averages of 5.30 and 131.61, respectively. Correlations were -0.040 for oral antidiabetic drugs and -0.112 for insulin.

**Conclusions:**

no correlation was found between the variables general comfort and medication adherence.

## RESUMEN

**Objetivos:**

comprobar la correlación hipotética entre el constructo de confort de la Teoría del Confort de Kolcaba y el constructo de adherencia al tratamiento con antidiabéticos orales e insulina en personas con Diabetes *Mellitus* tipo 2.

**Métodos:**

estudio transversal analítico con 198 participantes. Se midieron el confort y la adherencia a la medicación mediante el Cuestionario de Confort General, la Medida de Adherencia al Tratamiento con Insulina y la Medida de Adherencia al Tratamiento con Antidiabéticos Orales, respectivamente. Los datos se analizaron mediante estadística descriptiva e inferencial. Para el análisis de correlación se utilizó la prueba de correlación de Pearson.

**Resultados:**

la puntuación media de adherencia al tratamiento fue de 5,22 y la puntuación media de comodidad fue de 130,25 entre quienes usaban exclusivamente antidiabéticos orales, mientras que quienes usaban insulina obtuvieron medias de 5,30 y 131,61, respectivamente. Las correlaciones fueron de -0,040 para los antidiabéticos orales y de -0,112 para la insulina.

**Conclusiones:**

no se encontró correlación entre las variables comodidad general y adherencia al tratamiento.

## INTRODUCTION

In Diabetes *Mellitus* (DM) and other chronic diseases requiring prolonged treatment, both medication and non-medication adherence contribute to treatment success^(1)^, which favors metabolic control and avoids complications. Treatment adherence corresponds to the agreement between the guidance provided and patients’ own conduct, and is described from a multidimensional perspective^(2)^.

Adherence to non-pharmacological therapy in individuals with DM is related to lifestyle changes. Pharmacological therapy, on the other hand, is based on the use of oral antidiabetic drugs (OADs) or injectable medications, such as insulin, insulin analogs, and glucagon-like peptide-1 agonists. Various therapeutic combinations are also possible^(3)^.

Previous investigations point to factors that influence adherence to these therapies, such as bodily responses, medication side effects, knowledge about the health condition, cost of drug treatment, family support, patients’ relationship with the multidisciplinary team and health unit^(3,4)^. In this way, the aforementioned factors that influence adherence to drug treatment can be allocated to bodily, social, educational, and behavioral dimensions, characterizing the multidimensionality of the phenomenon.

From a theoretical-conceptual perspective, the authors of a broad systematic review identified one hundred and two conceptual models regarding factors that contribute to medication adherence in specific patient groups and/or specific diseases, applying the dimensions of factors related to patients, medication, the condition, the health system and professionals, and socioeconomic factors^(5)^. Among the models included, a holistic conceptual framework model was verified to describe medication adherence and guide DM interventions^(6)^. This holistic framework includes categories of factors which include elements of the comfort construct as a state in which basic needs are met for satisfaction, relief, and transcendence of people^(7,8)^.

Factors such as knowledge, quality of life, healthcare system resources, cultural perceptions, psychological feelings, and the interaction between caregiver and patient are some of the factors that can contribute to comfort from the perspective of physical, social, psycho-spiritual, and environmental contexts in Kolcaba’s Comfort Theory. The physical context consists of bodily sensations; the social context consists of belonging to interpersonal, family, and social relationships; the psycho-spiritual context encompasses the perception of the inner self, including self-esteem, concept, sexuality, meanings, and higher-order or being-related relationships; and the environmental context encompasses external actions of human experience, such as ambient colors, light, sound, temperature, and the influence of natural or synthetic elements^(7)^.

The General Comfort Questionnaire (GCQ) is an instrument used to measure comfort in four contexts, including being used to identify the effect of disease acceptance on the level of comfort in patients with type 2 DM^(9)^. Therefore, it has the potential to be an empirical reference for testing the theory from which it was derived.

The traditional approach to theoretical testing in nursing is hypothetico-deductive, meaning it produces a hypothesis in the form of a theoretical framework statement to be tested using statistical methods. These tests are most applicable to mid-range and micro-theories, which can be tested using descriptive, correlational, exploratory, and experimental designs depending on the theory’s purpose^(10)^.

From a hypothetical-deductive approach, a statement that the comfort offered by health professionals and systems would contribute to a patient’s decision to adhere to a therapeutic regimen^(5)^ could be used to investigate the association between comfort and adherence to treatments in people with DM, representing a test design of a relational statement between two constructs (comfort and adherence). The literature presents some relationships between comfort and discomfort linked to pain in insulin therapy^(11)^, describing emotional and psychological responses to the possibility of using insulin in conditions of insulin distress and diabetes distress^(12,13)^. Although such studies may capture variables related to comfort, they do not investigate the direct correlation between the construct of comfort and adherence to medication in people with type 2 DM.

### Study relevance

This article is the product of a graduation thesis(14) deposited in the institutional repository (https://pantheon.ufrj.br/handle/11422/16487).

## OBJECTIVES

To test a hypothetical correlation between the comfort construct of Kolcaba’s Comfort Theory and the adherence construct to OADs and insulin use in people with type 2 DM.

## METHODS

### Ethical aspects

The research project was previously assessed and authorized by the Research Ethics Committee in 2020. All participants were informed about the research and signed two copies of the Informed Consent Form, in accordance with Resolution 466/12.

### Theoretical framework

To provide a foundation for this study, Katharine Y. Kolcaba’s Comfort Theory was chosen as the central theoretical framework^(7)^. This is a mid-range theory, applicable in both research and nursing practice. In the Comfort Theory, Kolcaba conceptualizes comfort as a fundamental human need, manifesting in three distinct forms: satisfaction, relief, and transcendence^(7)^. These forms are achieved through nursing interventions, aimed at mitigating discomfort and promoting a person’s overall well-being.

Furthermore, the theory identifies four contexts where comfort is experienced: physical, psycho-spiritual, environmental, and sociocultural^(7)^. This structure facilitates the operationalization of the comfort construct in empirical studies, such as the present one, allowing for the measurement and correlation of comfort levels with relevant clinical variables.

The theoretical assumption adopted is that individuals with a higher perception of comfort (in its different forms and contexts) hypothetically exhibit greater adherence to pharmacological treatment (OADs and insulin). Thus, the theory was applied as a structuring framework for the design of study variables, their measurement, and the analysis of the correlation between comfort and medication adherence.

### Study design, period, and place

This is a cross-sectional analytical study with a quantitative approach, designed to test and discuss the plausibility of a theoretical hypothesis of correlation between two concepts or, more precisely, constructs. In empirical theoretical testing, correlation studies define the relationships between concepts described in the theory, but cannot identify causal relationships. Through a correlation coefficient, the type of relationship can be verified, however, without providing information about the structure of a theoretical model^(5)^. This observational study was reported based on the STrengthening the Reporting of OBservational studies in Epidemiology guidelines.

The study took place in a secondary-level outpatient service specializing in DM in the municipality of Macaé, Rio de Janeiro, Brazil. The facility provides care to pregnant women with diabetes, children, and adults with diabetes complications and/or difficulty managing blood glucose levels. Care is provided by a team consisting of an endocrinologist, psychologists, nutritionists, nurses, social workers, podiatrists, physiotherapists, and an occupational therapist.

Data were collected between September and December 2020 and February and March 2021. Field setup, participant recruitment, and data collection took place during activities related to medical and nursing consultations at the unit selected for the research. Service users who were scheduled for consultations during the data collection period were invited to participate in the study, based on general information about the research and the study methodology.

### Population and sample; inclusion and exclusion criteria

The study population consisted of individuals with type 2 DM undergoing drug treatment with OADs and/or insulin (monotherapy and/or combinations). Individuals aged 18 years or older, of both sexes, with type 2 DM and receiving treatment with OADs and/or insulin were included in the study. Individuals who were unable to maintain dialogue, understand, and/or verbalize, who had advanced chronic complications (amaurosis, amputees or wheelchair users, with chronic wounds and undergoing hemodialysis treatment), who had disabling health conditions such as heart failure (New York Heart Association ≥ 3), sequels of stroke, multiple sclerosis, Parkinson’s disease, irreversible and disabling paralysis with a functional independence measure (FIM) ≤103 points, or who were known to be pregnant were excluded. These criteria were assessed based on the potential participant’s clinical documentation. For potential participants with disabling health conditions, the FIM test was applied. And in individuals with heart failure, the symptoms present on the day of data collection were assessed to determine the functional classes of heart failure according to the New York Heart Association.

This study included 198 adults and older adults with type 2 DM. The sample size calculation was performed using the formula for cross-sectional correlational studies, adopting a 95% Confidence Interval (CI), with a significance level (Z) of 1.96, a sampling error (e) of 0.05 (5%), a high correlation of 0.80, and a test power of 80%, estimating a minimum sample size of 90 participants^(15)^. For this calculation, the Gpower3.1 software was used. The study employed non-probabilistic, convenience sampling, based on appointments scheduled during the data collection period.

### Study protocol

The research variables were categorized into socioeconomic, clinical, medication therapy, and study interest variables. The socioeconomic variables assessed were age, sex, education level, marital status, family income, and religion. The clinical and medication therapy variables assessed were average time to diagnosis, treatment description, medication class, and treatment duration. The variables of interest in this study were medication adherence measures, overall comfort, and its domains (physical, sociocultural, environmental, and psycho-spiritual).

For sample characterization, a specific instrument for socioeconomic and drug therapy assessment was used. Adherence to drug treatment was verified using the Insulin Treatment Adherence Measure (In Portuguese, *Medida de Adesão aos Tratamentos - insulina* - *MAT Insulina*) and the Oral Antidiabetic Treatment Adherence Measure (In Portuguese, *Medida de Adesão aos Tratamentos - antidiabéticos orais* - MAT ADO)^(16)^. These instruments are composed of seven items each, which present a response pattern ranging from “always” to “never”, with scores that can vary from one to six, respectively, and have face and criterion validity^(16)^. Medication adherence is determined by the overall average of the scores on the seven items of *MAT Insulina* and *MAT ADO*. Higher averages indicate greater adherence to the prescribed medication treatment^(16)^. For use in this research, permission was requested from the primary authors via email.

For comfort analysis, the authors used the Brazilian version of GCQ. This is a culturally adapted instrument validated for chronic kidney disease patients in Brazil^(17)^. The Brazilian version of GCQ contains 48 items that assess patient comfort in any clinical condition across physical, social, psycho-spiritual, and environmental dimensions. Each item on the questionnaire includes a four-point Likert scale, where one means patients completely disagree, and four means patients completely agree with the statement^(17)^. Scores range from 48 (very little comfort) to 192 (excellent comfort). It is a multidimensional instrument for identifying the different needs of patients. Reliability was shown, verified by Cronbach’s alpha of 0.80, with the 48 items of the scale varying between 0.791 and 0.818^(17)^. For its use in this research, authorization was also requested via email.

The four data collection instruments were applied in a single meeting with each participant, lasting an average of 30 minutes. The sequence consisted of a sociodemographic and clinical characterization instrument, *MAT Insulina* and *MAT ADO*, and finally, the GCQ application. The questions and answers were verbalized by participants and completed by the research team.

### Analysis of results and statistics

The data were analyzed using descriptive and inferential statistics, using the Statistical Package for the Social Sciences version 20.0. Measures of position (mean, minimum, and maximum) and dispersion (standard deviation) were calculated, using Pearson’s correlation test to analyze the correlation between the variables of medication adherence, general comfort, and comfort domains. A 95% CI was applied.

The analysis considered two groups: 1) Exclusive use of OAD; and 2) Use of insulin. Due to limitations of the medication adherence instruments used in this investigation, it was not possible to consider the concomitant use of OAD and insulin. Therefore, in cases where participants used both OADs and insulin, only the insulin adherence instrument was completed. Although it is recognized that this analysis does not verify medication adherence in people using both OADs and insulin, the organization between these two groups can be justified by the clinical and therapeutic aspects for people with type 2 DM. It is understood that insulin therapy is only indicated in symptomatic type 2 DM individuals with glycated hemoglobin greater than 9%, or for therapeutic intensification in asymptomatic type 2 DM individuals with HbA1c between 7.5% and 9% after three months of dual combination therapy with OAD. Therefore, using insulin alone or in combination with OADs clinically presents a challenge in maintaining individual glycemic targets for people with DM, thus justifying the prioritization of insulin adherence assessment.

It is noteworthy that, during the data analysis period, multiple linear regression was attempted. However, because the study did not define the sample size according to the finite population for multiple linear regression, considering the number of independent and significant variables in each treatment group, certain assumptions would be violated. Moreover, in an analysis performed after data collection, only two participants met all criteria equally for each independent variable. Another assumption that limited the use of logistic regression was the residuals, as this study presented abnormally distributed residuals.

## RESULTS

The sample consisted of 198 people with type 2 DM. Participants were predominantly female (66.20%), with a mean age of 62.50 (SD=11.06) years, marital status married (45.50%), considered evangelical (61.60%), with incomplete elementary school (43.40%), and a mean family income of one to two minimum wages (66.20%).

Concerning clinical and medication treatment variables, the mean time since diagnosis of type 2 DM ranged from 0.2 to 38.0 years, with a mean of 16.40 years (SD=7.65). The mean treatment time ranged from 0.2 to 30 years, with a mean of 8.84 years (SD=5.55). Characterization of treatment regimen among participants showed that the majority OADs and insulin (50.01%), followed by those using only OADs (36.36%) and those using only insulin (13.63%).

In relation to the combination of medications in the treatment regimen, it was observed that the majority of participants who had a treatment regimen with OADs and insulin used a triple combination (40.47%) of drug classes. Of the participants who use only insulin, 55.55% use a combination of insulin and analogues, and among participants who use only OADs, the double combination of drug classes was the most prevalent (43.05%).

**Figure 1:**
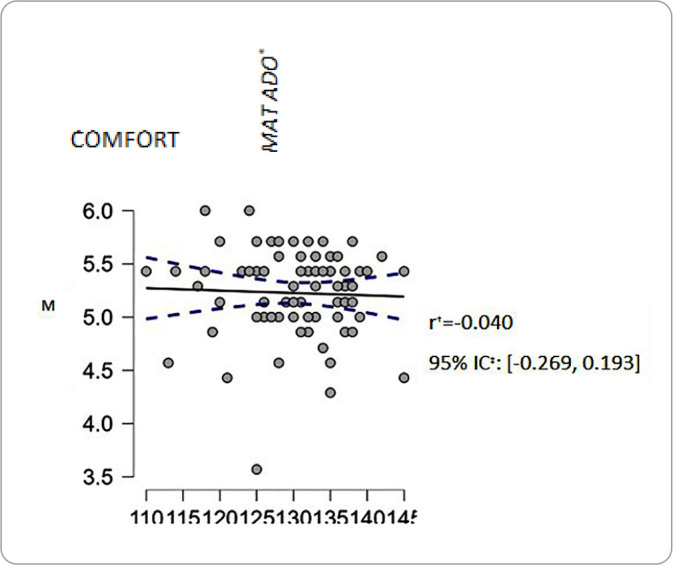
Correlation between overall comfort and medication adherence in participants (N=72) who exclusively use oral antidiabetic drugs, Macaé, Rio de Janeiro, Brazil, 2021

Seventy-two *MAT ADO* and 126 *MAT Insulina* instruments were completed. Regarding *MAT ADO*, the mean score was 5.22 (SD=0.41), while *MAT Insulina* instrument had a mean score of 5.30 (SD=0.34). The mean comfort score for participants using only OADs was 130.25 (SD=7.37), while for those using insulin it was 131.61 (SD=6.77). The correlation between the variables of general comfort and adherence among participants using only OADs was null (Figure 1).

*MAT ADO - Medida de Adesão aos Tratamentos - antidiabéticos orais; †r - Pearson’s rho; ‡CI - Confidence Interval.

**Figure 2:**
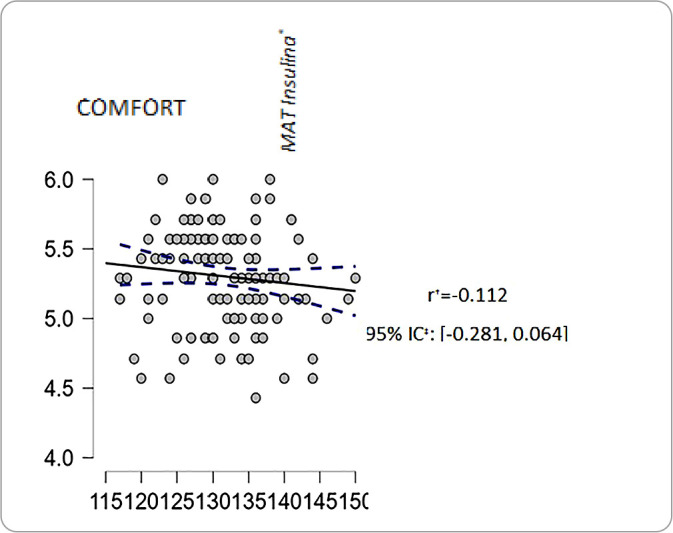
Correlation between overall comfort and medication adherence in participants (N=126) using insulin, Macaé, Rio de Janeiro, Brazil, 2021

Similarly, the correlation between the variables general comfort and adherence among participants using insulin was found to be null, as can be seen in Figure 2.

*MAT ADO - Medida de Adesão aos Tratamentos - antidiabéticos orais; ††r - Pearson’s rho; ‡CI - Confidence Interval.

Correlation analysis between medication adherence variables and comfort domains in participants using oral contraceptives found no statistically significant difference. In participants using insulin, no statistically significant correlations were observed either. Table 1 shows the details.

**Table 1:** Correlation between comfort dimensions and adherence among those exclusively using oral antidiabetic drugs (n=72) and those using insulin (n=127), Macaé, Rio de Janeiro, Brazil, 2021

Pearson’s rho	Physical	Sociocultural	Environmental	Psycho-spiritual
Correlations of *MAT ADO* ^^*^^	-0.052	-0.155	0.014	-0.078
*p* **^†^**	0.561	0.083	0.877	0.384
Correlations of *MAT Insulina* ^‡^	-0.019	-0.157	-0.018	0.076
*p* **^†^**	0.875	0.188	0.879	0.523

* *MAT ADO - Medida de Adesão aos Tratamentos - antidiabéticos orais; †p - Significance level; ‡MAT Insulina - Medida de Adesão aos Tratamentos - insulina.*

## DISCUSSION

The main results of the study indicated a sample of participants with good medication adherence for both insulin users and users of OADs. Regarding comfort, scores on GCQ in the 130-point range for both groups indicate a good level of comfort. In the correlation analysis, there was no association between overall comfort and medication adherence to OADs and insulin.

As for the results of good medication adherence identified in this investigation, the literature indicates a need to develop a consensus on what constitutes good medication adherence in diabetes. A systematic review identified a high divergence in the prevalence rate of medication adherence to insulin and/or oral antidiabetic drugs (38.5% to 93.1%), with only six of the 27 studies (22.2%) reporting a prevalence of adherence ≥ 80% among the studied population^(18,19)^. A recent review, focused solely on the use of OADs, identified a pooled proportion of adherent patients of 54% (95%CI, CI: 51-58%), demonstrating that adherence to OADs in patients with type 2 DM is suboptimal^(20)^.

A possible reason for the high level of medication adherence found may be associated with the group being characterized by a majority of elderly individuals. A systematic review with meta-analysis on sociopersonal factors affecting adherence showed that younger individuals have a 17% higher risk of non-adherence to treatment than older individuals^(21)^.

From an empirical standpoint, sociocultural and psycho-spiritual issues are related to treatment adherence. Family support was a major facilitator for adherence to medication treatment in type 2 DM, while the cost of medications and poor communication with prescribers were barriers^(22)^. In the psycho-spiritual context, positive emotions, such as empowerment and the ability to continue with self-care in light of the benefits of treatments, and negative emotions, such as fear, self-blame, guilt, helplessness, frustration, and worry about the potential complications of diabetes, influence adherence to medication treatment in type 2 DM^(23)^. These emotions are linked to psycho-spiritual perspectives. Evidence indicates that spiritual intelligence and emotional intelligence can complement each other to increase self-management and adaptive mechanisms in people with diabetes^(24)^, or benefit the patient through spirituality and religiosity, including in coping with stressful situations caused by the disease conditions^(25)^.

The relationship between comfort, another variable identified as having a good level, and medication adherence characterizes the originality of this investigation. No similar investigations were found in the literature. In parallel, a study conducted in Turkey assessed the comfort level of people with type 2 DM using GCQ and the relationship between comfort and acceptance of the disease^(9)^. A weak positive correlation was detected between the Acceptance of Illness Scale and the mean scores of GCQ and its subdimensions. In the investigation^(9)^, the mean comfort levels were not presented. However, education, income, profession, people with whom patients lived, frequency of follow-up, practice of diets and physical exercise, and chronic complications were considered significant predictors for the overall comfort level.

In parallel with the identified predictors^(9)^ and the variables investigated in the participants of this research, we can hypothesize reasons why the detected comfort level was high. Among the predictors, we highlight the level of education (low education level identified) and income (low income identified). The literature understands that a high level of education reduces comfort levels, because the more information, the greater the chance of worrying about the prognosis of the disease and increasing the psychological burden^(9)^. Although higher incomes increase expectations regarding illness and may negatively affect comfort, low income levels are not sufficient to meet those expectations^(9)^. In contrast to these highlighted predictors of comfort, when considering these characteristics in relation to treatment adherence, education level and income show both positive and negative relationships in studies retrieved by systematic reviews, a characteristic of the heterogeneity of research conducted in this area^(21,26)^.

Based strictly on the evidence from this investigation, the relational assertion that a person, upon achieving comfort through some therapeutic intervention, has an increased likelihood of engaging in health-seeking behaviors is not supported^(7,8)^. However, before abandoning any interest in the proposition and the theory as a whole, we affirm that the findings are discussed in light of the assertion that “general knowledge is theoretical knowledge”. Through their abstraction, theories are applied to specific locations and populations, but empirical findings are not^(19)^.

Researchers typically assess the possibility of generalizing the findings obtained in a particular study to other populations or groups of people, other times, places, contexts, or other categories of unique yet similar situations, as a criterion for the usefulness of their research^(19,27)^. In research designed for theoretical testing, the targets to be tested may be abstract concepts, such as comfort and adherence. Therefore, external validity must be considered in relation to this more abstract type of target^(28)^.

If we consider the instruments for measuring the concepts of comfort and adherence (GCQ and *MAT*) to be adequate, problems would remain regarding the research design and/or deficiencies in the theory being tested through its constructs. Firstly, we assume that there is a limitation in the methodological design stemming from a failure to observe a formulation of the theory that underpinned the construction of GCQ, thus pointing to an illusory deficiency in the plausibility of the theory.

GCQ, as a theory-derived instrument, represents an attempt to bring assertions and other elements of Kolcaba’s Comfort Theory into the empirical and operational domain. Although the scope of this transfer from theory to measurement can always be debated, the scales are applied from the perspective that aspects of the theory are incorporated into them. Therefore, although GCQ does not explicitly state this, it presumes the existence of a theoretical definition for the concept of comfort. Considering the four contexts (physical, psycho-spiritual, environmental, and sociocultural), comfort would be a dynamic state, subject to rapid positive and negative changes^(28)^.

Therefore, if the state of comfort is indeed dynamic and subject to rapid changes, as the theoretical definition suggests, then a cross-sectional application of the measurement instrument would not be able to measure the correlation that should be accompanied by a longitudinal method design. Thus, if, on the one hand, the research planning did not contain a methodological contraindication to applying the instrument cross-sectionally to investigate, through statistical means, the existence of a correlation, on the other hand, the non-observance of the definition of comfort, a non-relational theoretical statement, may be a good explanation for not finding the correlation that was established in the hypothesis, even if it may exist.

For a more radical empiricist, the limitations of the methodological design associated with the nullity of correlation would be sufficient to end the debate about any possible relevance of this research. However, science does not always advance in directly successful cases. Results identified in another research group support this contradiction. Research conducted among adolescents with type 1 DM identified, through logistic regression, that the highest level of self-reported comfort with diabetes management tasks was associated with higher glycated hemoglobin (p=0.006), after controlling for age, sex, race, type of health insurance, and duration of diabetes^(29)^. It is noteworthy that self-reported comfort with independent self-management of type 1 DM may not be a sufficient metric to assess the need for further intervention to optimize glycemic outcomes in the transition from adolescence to adulthood^(29)^. There are no apparent methodological limitations in the aforementioned research^(29)^. However, once again, theory informs aspects for the construction of general knowledge, modulating issues of external validity.

When a theoretical proposition of an expected relationship between two concepts is not verified by study under natural conditions, two considerations may arise *a priori:* the test has low external validity, or if the test is well-designed, there are factors between the concepts that the theory does not specify. Thus, explicitly, “if experimental tests can handle all theoretically significant variables and nothing else, then a problem with the external validity of an experiment is a problem with the usefulness of the theory”^(19)^.

### Study limitations

This observational study was directly impacted by the difficulty in locating the critical theoretical statements for the theoretical test, because the theoretical definition employed for the concept of comfort in the construction of GCQ and its cultural adaptation for Brazil omitted this property of temporal dynamism^(17)^, with the consideration as a dynamic state appearing only in one publication directed at perianesthesia situations^(28)^. Thus, the cross-sectional methodological design disregarded the temporal specificity of the concept to be measured, pointing to a limitation of the isolated application of GCQ, especially in theoretical testing studies.

Furthermore, the non-probabilistic selection of the analyzed sample is noteworthy. Additionally, the study considered the healthcare setting, disregarding the environment in which participants receive their medication treatment, whether at home or at work. Another limitation was the inability to verify adherence among those using both insulin and OADs, since the instrument was separate, preventing a joint analysis of individuals using both medication regimens. Therefore, only insulin was analyzed in these cases.

### Contributions to nursing

The results of this research expand the body of nursing knowledge by investigating, for the first time in the Brazilian national context, the relationship between the construct of comfort and medication adherence in people with type 2 DM. Although no statistically significant correlation was observed among variables, the findings offer important insights for practice, highlighting the levels of comfort perceived by the participants and the pattern of adherence to the use of OADs or insulin.

These findings contribute to critical reflection on factors that influence adherence to treatment, reinforcing the need for multifactorial approaches that include, in addition to comfort, aspects such as social support, health education, motivation, and management of individual and collective barriers. From a theoretical point of view, the results provide elements for refining the application of Kolcaba’s Comfort Theory in studies focused on health behaviors, encouraging new research that explores interactions with other clinical, psychosocial, and cultural variables, as well as their temporality.

In the context of care, the study indicates that nursing care planning should consider comfort as an essential component for well-being and quality of life, but that its isolated influence on adherence may be limited, requiring integrated and personalized interventions.

## CONCLUSIONS

Although good overall comfort and medication adherence were observed among the research participants, the hypothetical correlation between comfort according to Kolcaba’s theory and the medication adherence construct appears to have been refuted, as no correlation was found between the variables. However, our theoretical discussion concluded that the definition of the comfort construct, derived from Kolcaba’s theory, portrays a dynamic state subject to rapid changes. This may explain the lack of correlation between the constructs in cross-sectional research, which does not refute the hypothetical existence of a correlation from a longitudinal perspective. Thus, future studies can advance knowledge to elucidate the theoretical hypothesis.

## Data Availability

The research data are available within the article.
