# Supplementary material for: Correlation between comfort and medication adherence in people with type 2 Diabetes Mellitus: a cross-sectional study
Source: Rev Bras Enferm. 2026 Jul 17;79:e20240449. doi: 10.1590/0034-7167-2024-0449 (PMC13379195; doi:10.1590/0034-7167-2024-0449)
Supplement: Translation

# Translation: Correlação entre conforto e adesão medicamentosa de pessoas com Diabetes *Mellitus* tipo 2: estudo transversal

**DOI:** 10.1590/0034-7167-2024-0449pt

**Authors:** Rafael Oliveira Pitta Lopes, Simone Paulino Ferreira Coimbra, Hudson Carmo de Oliveira, Juliana Faria Campos, Renan Alves Silva, Marcos Antônio Gomes Brandão

**Affiliations:** I Universidade Federal do Rio de Janeiro. Rio de Janeiro, Rio de Janeiro, Brasil; II Universidade Federal do Rio de Janeiro. Macaé, Rio de Janeiro, Brasil; III Universidade Federal de Campina Grande. Cajazeiras, Paraíba, Brasil

**Keywords:** Adesão à, Medicação, Conforto do Paciente, Diabetes *Mellitus* Tipo 2, Teoria de Enfermagem, Insulina.

## Abstract

**Objetivos:**

testar uma hipotética correlação entre o construto conforto da Teoria do Conforto de Kolcaba e o construto adesão ao uso de antidiabéticos orais e insulina em pessoas com Diabetes *Mellitus* tipo 2.

**Métodos:**

estudo transversal analítico, realizado com 198 participantes. O conforto e adesão medicamentosa foram mensurados, respectivamente, pelos instrumentos Questionário de Conforto Geral, Medida de Adesão aos Tratamentos - insulina e Medida de Adesão aos Tratamentos - antidiabéticos orais. Os dados foram analisados por estatística descritiva e inferencial. Para análise de correlação, utilizou-se o teste de correlação de Pearson.

**Resultados:**

a média de adesão medicamentosa foi de 5,22, e a média de conforto, de 130,25 entre os que utilizavam exclusivamente antidiabéticos orais, enquanto os que faziam insulina apresentaram médias de 5,30 e 131,61. As correlações foram -0,040, para antidiabéticos orais, e -0,112, para insulina.

**Conclusões:**

foi verificada correlação nula entre as variáveis conforto geral e adesão medicamentosa.

## INTRODUÇÃO

No Diabetes *Mellitus* (DM) e em outras doenças crônicas que exigem um tratamento prolongado, tanto a adesão medicamentosa quanto a não medicamentosa contribuem para o sucesso do tratamento^(1)^, o que favorece o controle metabólico e evita complicações. A adesão ao tratamento corresponde à concordância entre a orientação repassada e a conduta do próprio paciente, sendo descrita em uma perspectiva multidimensional^(2)^.

A adesão à terapia não medicamentosa da pessoa com DM está relacionada às mudanças no estilo de vida. Já a terapia medicamentosa está pautada no uso de antidiabéticos orais (ADOs) ou medicamentos injetáveis, como a insulina, análogos e os agonistas do peptídeo semelhante ao glucagon-1. São possíveis, ainda, diversas combinações terapêuticas^(3)^.

Investigações anteriores apontam fatores que influenciam a adesão destas terapêuticas, tais como respostas corporais, efeitos colaterais dos medicamentos, conhecimento sobre a condição de saúde, custo com tratamento medicamentoso, apoio familiar, vínculo do paciente com a equipe multiprofissional e unidade de saúde^(3,4)^. Dessa maneira, os mencionados fatores que influenciam a adesão do tratamento medicamentoso podem ser alocados em dimensões corporais, sociais, educacionais e comportamentais, caracterizando a multidimensionalidade do fenômeno.

Na perspectiva teórico-conceitual, os autores de ampla revisão sistemática identificaram cento e dois modelos conceituais sobre fatores que contribuem na adesão medicamentosa de grupos de pacientes específicos e/ou doenças específicas, aplicando as dimensões de fatores relacionados ao paciente, à medicação, à condição, ao sistema de saúde e profissionais, e aos fatores socioeconômicos^(5)^. Entre os modelos incluídos, foi verificado um modelo de estrutura conceitual holística para descrever a adesão à medicação e orientar intervenções em DM^(6)^. Essa estrutura holística inclui categorias de fatores nos quais estão incluídos elementos do construto conforto como estado no qual as necessidades básicas são atendidas para satisfação, alívio e transcendência da pessoa^(7,8)^.

Fatores como conhecimento, qualidade de vida, recursos relacionados ao sistema de saúde, percepções culturais, sentimentos psicológicos, e interação entre provedor do cuidado e paciente são alguns fatores que podem compor o conforto na perspectiva dos contextos físico, social, psicoespiritual e ambiental na Teoria do Conforto de Kolcaba. O contexto físico consiste nas sensações corporais; o contexto social consiste no pertencimento a relações interpessoais, familiares e sociais; o contexto psicoespiritual abrange a percepção do eu interior, incluindo estima, conceito, sexualidade, significados e relações de alta ordem ou de estar; e o contexto ambiental engloba ações externas da experiência humana, como cores do ambiente, luz, som, temperatura e influência de elementos naturais ou sintéticos^(7)^.

O Questionário de Conforto Geral (QCG) é um instrumento utilizado para medir o conforto nos quatro contextos, inclusive sendo utilizado para identificar o efeito da aceitação da doença no nível de conforto em pacientes com DM tipo 2^(9)^. Sendo assim, tem potencial de ser um referente empírico para um teste da teoria do qual foi derivado.

A abordagem tradicional do teste teórico na enfermagem é a hipotético-dedutiva, ou seja, a que produz a hipótese na forma de afirmativa de estrutura teórica para ser testada por métodos estatísticos. Os testes são mais aplicáveis a teorias de médio alcance e microteorias, que podem ser testadas por desenhos descritivos, correlacionais, exploratórios e experimentais em função do tipo de finalidade da teoria^(10)^.

A partir de uma abordagem hipotético-dedutiva, uma afirmativa de que o conforto oferecido por profissionais e sistema de saúde contribuiria para a decisão de um paciente em aderir a regime terapêutico^(5)^ poderia ser usada para investigar a associação entre conforto e adesão a tratamentos em pessoas com DM, representando um delineamento de testagem de uma afirmativa relacional entre dois construtos (conforto e adesão). A literatura apresenta algumas relações entre conforto e desconforto ligadas à dor na terapia com insulina^(11)^, descrevendo respostas emocionais e psicológicas diante da possibilidade de uso de insulina nas condições de *insuline distress* e *diabetes distress* ^(12,13)^. Embora tais estudos possam apreender variáveis relacionadas ao conforto, eles não investigam a correlação direta entre o construto conforto e a adesão a formas medicamentosas em pessoas com DM tipo 2.

### Relevância do estudo

Este artigo é produto de trabalho de conclusão de graduação^(14)^ depositado no repositório institucional (https://pantheon.ufrj.br/handle/11422/16487).

## OBJETIVOS

Testar uma hipotética correlação entre o construto conforto da Teoria do Conforto de Kolcaba e o construto adesão ao uso de ADOs e insulina em pessoas com DM tipo 2.

## MÉTODOS

### Aspectos éticos

O projeto de pesquisa foi previamente avaliado e autorizado pelo Comitê de Ética em Pesquisa no ano de 2020. Todos os participantes foram informados sobre a pesquisa, e assinaram duas vias do Termo de Consentimento Livre e Esclarecido, estando em conformidade com a Resolução n^o^ 466/12.

### Referencial teórico

Para fundamentar o presente estudo, optou-se por utilizar como referencial teórico central a Teoria do Conforto de Katharine Y. Kolcaba^(7)^. Trata-se de teoria de médio alcance, aplicável em contexto de pesquisa e da prática de enfermagem. Na Teoria do Conforto, Kolcaba conceitua o conforto como uma necessidade humana fundamental, que se manifesta em três formas distintas: satisfação, alívio e transcendência^(7)^. Essas formas são alcançadas por meio de intervenções de enfermagem, visando mitigar o desconforto e promover o bem-estar integral da pessoa.

Além disso, a teoria identifica quatro contextos onde o conforto é experienciado: físico, psicospiritual, ambiental e sociocultural^(7)^. Essa estrutura facilita a operacionalização do construto conforto em estudos empíricos, como o presente, permitindo mensurar e correlacionar níveis de conforto com variáveis clínicas relevantes.

O pressuposto teórico adotado é que indivíduos com maior percepção de conforto (em suas diferentes formas e contextos) apresentam, de forma hipotética, maior adesão ao tratamento farmacológico (ADOs e insulina). Assim, a teoria foi aplicada como referencial estruturante para o delineamento das variáveis do estudo, sua mensuração, e a análise da correlação entre conforto e adesão medicamentosa.

### Desenho, período e local do estudo

Trata-se de estudo do tipo transversal analítico de abordagem quantitativa, para testar e discutir a plausabilidade de uma hipótese teórica de correlação entre dois conceitos ou, mais precisamente, construtos. No teste teórico empírico, os estudos de correlação definem as relações entre conceitos descritos na teoria, mas não podem identificar relações de natureza causal. Por meio de um coeficiente de correlação, pode-se verificar o tipo de relação, entretanto, sem informar sobre a estrutura de um modelo teórico^(5)^. Este estudo observacional foi relatado a partir das diretrizes *STrengthening the Reporting of OBservational studies in Epidemiology*.

O estudo ocorreu em serviço ambulatorial, de nível secundário, especializado em DM do município de Macaé, Rio de Janeiro, Brasil. No local, são oferecidos atendimentos a gestantes com diabetes, crianças e adultos com complicações do diabetes e/ou com dificuldade no manejo glicêmico. O atendimento é realizado por uma equipe formada por médico endocrinologista, psicólogos, nutricionistas, enfermeiros, assistentes sociais, podólogos, fisioterapeutas e terapeuta ocupacional.

Os dados foram coletados nos períodos de setembro a dezembro de 2020 e fevereiro a março de 2021. A ambiência ao campo, recrutamento dos participantes e processo de coleta de dados ocorreram durante atividades ligadas às consultas médicas e de enfermagem, realizadas na unidade selecionada para a pesquisa. Os usuários dos serviços que estavam agendados para consulta no período de coleta de dados foram convidados a integrar o estudo, a partir de informações gerais sobre a pesquisa e o método de estudo.

### População e amostra; critérios de inclusão e exclusão

A população considerada foi pessoas com DM tipo 2 em tratamento medicamentoso por ADOs e/ou insulina (monoterapia e/ou associações). Foram incluídas no estudo pessoas com idade igual ou maior a de 18 anos, de ambos os sexos, com DM tipo 2 e que realizam tratamento com ADOs e/ou insulina. Foram excluídas pessoas que possuíam incapacidade de manter o diálogo, de compreensão e/ou verbalização, que apresentem complicações crônicas em estágio avançado (amaurose, amputados ou cadeirantes, com feridas crônicas e que estejam em tratamento hemodialítico), tenham condições de saúde incapacitantes como insuficiência cardíaca (*New York Heart Association* ≥ 3), sequelas de acidente vascular cerebral, esclerose múltipla, doença de Parkinson, paralisia irreversível e incapacitante com medida de independência funcional (MIF) ≤103 pontos ou que sabidamente estejam em período gestacional. Esses critérios foram avaliados na documentação clínica do potencial participante. Nos potenciais participantes com condições de saúde incapacitantes, foi aplicado o teste de MIF. E em pessoas com insuficiência cardíaca, foram avaliados os sintomas presentes no dia da coleta de dados para determinar as classes funcionais da insuficiência cardíaca de acordo com a *New York Heart Association*.

Participaram deste estudo 198 adultos e idosos com DM tipo 2. O cálculo amostral se deu pela fórmula para estudos transversais correlacionais, adotando o Intervalo de Confiança (IC) de 95%, com nível de significância (Z) de 1,96, erro amostral (e) de 0,05 (5%), correlação alta de 0,80 e um poder de teste de 80%, estimando uma amostra mínima de 90 participantes^(15)^. Para este cálculo, foi utilizado o *software* Gpower3.1. O estudo empregou amostragem não probabilística, por conveniência, de acordo com os atendimentos agendados no período de coleta de dados.

### Protocolo do estudo

As variáveis da pesquisa foram categorizadas em variáveis socioeconômicas, clínicas, de terapia medicamentosa e de interesse do estudo. As variáveis socioeconômicas avaliadas foram idade, sexo, escolaridade, estado civil, renda familiar e religião. As variáveis clínicas e de terapia medicamentosa avaliadas foram tempo médio de diagnóstico, descrição do tratamento, classe medicamentosa e tempo de tratamento. As variáveis de interesse deste estudo foram medida de adesão medicamentosa, conforto geral e seus domínios (físico, sociocultural, ambiental e psicoespiritual).

Para caracterização da amostra, foi utilizado instrumento próprio de avaliação socioeconômica e de terapia medicamentosa. A verificação da adesão ao tratamento medicamentoso foi feita pela Medida de Adesão aos Tratamentos - insulina (MAT Insulina) e Medida de Adesão aos Tratamentos - antidiabéticos orais (MAT ADO)^(16)^. Esses instrumentos são compostos por sete itens cada, que apresentam um padrão de resposta que vai de “sempre” até “nunca”, com escores que podem variar de um a seis, respectivamente, e possuem validação de face e de critério^(16)^. A adesão medicamentosa é determinada pela média global dos escores dos sete itens do MAT Insulina e MAT ADO. Médias mais altas indicam maior adesão ao tratamento medicamentoso prescrito^(16)^. Para utilização nesta pesquisa, foi solicitada autorização aos autores primários por meio de correio eletrônico.

Para análise do conforto, os autores usaram a versão brasileira do QCG. Trata-se de instrumento adaptado culturalmente e validado para pacientes renais crônicos no Brasil^(17)^. A versão brasileira do QCG contém 48 itens, que avaliam o conforto de pacientes em qualquer condição clínica nas dimensões físicas, sociais, psicoespirituais e ambientais. Cada item do questionário inclui uma escala tipo Likert de quatro pontos, na qual um significa que o paciente discorda completamente, e quatro, que o paciente concorda completamente com a afirmação^(17)^. Os escores variam de 48 (muito pouco conforto) até 192 (excelente conforto). É um instrumento multidimensional para identificação das diferentes necessidades dos pacientes. Foi apresentada confiabilidade verificada pelo alfa de Cronbach de 0,80, variando os 48 itens da escala entre 0,791 e 0,818^(17)^. Para utilização nesta pesquisa, também foi solicitada autorização por meio de correio eletrônico.

Os quatro instrumentos de coleta de dados foram aplicados em um mesmo encontro com cada participante, com duração média de 30 minutos, sendo a sequência instrumento de caracterização sociodemográfica e clínica, MAT Insulina e/ou MAT ADO, e, por fim, aplicação do QCG. Os questionamentos e as respostas eram verbalizados pelo participante e preenchidas pela equipe de pesquisa.

### Análise dos resultados e estatística

Os dados foram analisados por estatística descritiva e inferencial, na qual foi utilizado o *Statistical Package for the Social Sciences*, versão 20.0. Foram calculadas as medidas de posição (média, mínima e máxima) e de dispersão (desvio padrão), utilizando o teste de correlação de Pearson para análise da correlação entre as variáveis de adesão medicamentosa, conforto geral e domínios de conforto. O IC aplicado foi de 95%.

A análise levou em consideração dois grupos: 1) Uso exclusivo de ADOs; e 2) Uso de insulina. Por limitação dos instrumentos relacionados à adesão medicamentosa utilizados nesta investigação, não foi possível considerar o uso concomitante do ADO e insulina. Dessa forma, em casos em que o participante fazia uso associado de ADOs e insulina, era preenchido apenas o instrumento de adesão à insulina. Apesar de reconhecer que esta análise não verifica a adesão medicamentosa em pessoas com associação entre ADO e insulina, a organização entre esses dois grupos pode ser justificada pelo aspecto clínico e terapêutico para pessoas com DM tipo 2. Compreende-se que a terapia com insulina só é indicada em pessoas com DM tipo 2 sintomáticas com hemoglobina glicada superior a 9% ou para intensificação terapêutica de pessoas com DM tipo 2 assintomáticas com hemoglobina glicada entre 7,5 e 9% após três meses de terapia de associação dupla com ADO. Assim, fazer uso de insulina de forma isolada ou associada a ADOs representa clinicamente uma dificuldade na manutenção das metas glicêmicas individuais de pessoas com DM tipo 2, justificando, portanto, a priorização da avaliação de adesão à insulina.

Destaca-se que, no período de análise de dados, buscou-se operacionalizar a regressão linear múltipla, entretanto, pelo estudo não ter definido o tamanho da amostra de acordo com a população finita para regressão linear múltipla, considerado o número de variáveis independentes e significativas em cada um dos grupos de tratamento, pressupostos seriam feridos. Em adição, em análise realizada após coleta, apenas dois participantes apresentavam todos os critérios respondidos por igual em cada variável independente. Outro pressuposto que limitou a realização de regressão logística foram os resíduos da regressão, tendo este estudo apresentado resíduos de distribuição anormal.

## RESULTADOS

A amostra foi composta por 198 pessoas com DM tipo 2. Os participantes predominantemente foram pessoas do sexo feminino (66,20%), com média de idade de 62,50 (DP=11,06) anos, estado civil casado (45,50%), considerados evangélicos (61,60%), escolaridade de ensino fundamental incompleto (43,40%), e renda familiar média de um a dois salários mínimos (66,20%).

Em relação às variáveis clínicas e de tratamento medicamentoso, o tempo médio de diagnóstico de DM tipo 2 variou de 0,2 a 38 anos, com média de 16,40 anos (DP=7,65). O tempo médio de tratamento variou de 0,2 a 30 anos, com média de 8,84 anos (DP=5,55). A caracterização do regime de tratamento entre os participantes constatou que a maioria fazia uso de ADOs e insulina (50,01%), seguida dos que usavam apenas ADOs (36,36%) e os que usavam apenas insulina (13,63%).

No que tange ao esquema de associações do regime de tratamento medicamentoso, observou-se que a maioria dos participantes que possuíam o regime de tratamento com ADOs e insulina realizava tripla associação (40,47%) de classes medicamentosas. Dos participantes que utilizam somente insulina, 55,55% fazem associação da insulina e análogos, e entre os participantes que utilizam apenas ADOs, a dupla associação de classes medicamentosas foi a mais prevalente (43,05%).

Foram preenchidos 72 instrumentos MAT ADO e 126 MAT Insulina. Em relação ao instrumento MAT ADO, a média dos escores foi de 5,22 (DP=0,41), enquanto o instrumento MAT Insulina obteve média de 5,30 (DP=0,34). A média do conforto dos participantes em uso exclusivo de ADO foi de 130,25 (DP=7,37), enquanto os que faziam insulina foi de 131,61 (DP=6,77). A correlação entre as variáveis de conforto geral e adesão entre os participantes que faziam uso exclusivo de ADOs se mostrou nula (Figura 1).

**Figura 1:** Correlação entre conforto geral e adesão medicamentosa nos participantes (N=72) que fazem uso exclusivo de antidiabéticos orais, Macaé, Rio de Janeiro, Brasil, 2021

*MAT ADO - Medida de Adesão aos Tratamentos - antidiabéticos orais; †r - Ró de Pearson; ‡IC - Intervalo de Confiança.

Do mesmo modo, a correlação entre as variáveis conforto geral e adesão entre os participantes em uso de insulina se mostrou nula, como é possível observar na Figura 2.

**Figura 2:** Correlação entre conforto geral e adesão medicamentosa nos participantes (N=126) que fazem uso de insulina, Macaé, Rio de Janeiro, Brasil, 2021

*MAT ADO - Medida de Adesão aos Tratamentos - antidiabéticos orais; †r - Ró de Pearson; ‡IC - Intervalo de Confiança.

A análise de correlação entre as variáveis de adesão medicamentosa e os domínios de conforto nos participantes que utilizavam ADOs não encontrou diferença estatística. Nos participantes em uso de insulina, também se observou ausência de correlações estatísticas. Detalhes são observados na Tabela 1.

**Tabela 1:** Correlação entre as dimensões de conforto com adesão daqueles que fazem uso exclusivo de antidiabéticos orais (n=72) e aqueles em uso de insulina (n=127), Macaé, Rio de Janeiro, Brasil, 2021

Ró de Pearson	Físico	Sociocultural	Ambiental	Psicoespiritual
Correlações da MAT ADO^^*^^	-0,052	-0,155	0,014	-0,078
*p* **^†^**	0,561	0,083	0,877	0,384
Correlações da MAT Insulina^‡^	-0,019	-0,157	-0,018	0,076
*p* **^†^**	0,875	0,188	0,879	0,523

* *MAT ADO - Medida de Adesão aos Tratamentos - antidiabéticos orais; †p - Nível de significância; ‡MAT Insulina - Medida de Adesão aos Tratamentos - insulina.*

## DISCUSSÃO

Os principais resultados do estudo indicaram uma amostra de participantes com boa adesão medicamentosa tanto para os usuários de insulina quanto para os usuários de ADOs. No nível de conforto, os valores no QCG na faixa dos 130 pontos para ambos os grupos indicam bom nível de conforto. Na análise de correlação, a associação foi nula entre conforto geral e adesão medicamentosa a ADOs e insulina.

No que se refere aos resultados de boa adesão medicamentosa identificada nesta investigação, consta-se na literatura a necessidade de desenvolver consenso sobre o que constitui uma boa adesão medicamentosa ao diabetes. Revisão sistemática identificou alta divergência na taxa de prevalência da adesão medicamentosa ao uso de insulina e/ou ADOs (38,5 a 93,1%), em que apenas seis dos 27 estudos (22,2%) relataram prevalência de adesão ≥ 80% entre a população estudada^(18,19)^. Revisão recente, focada apenas ao uso de ADOs, identificou uma proporção agrupada de pacientes aderentes de 54% (IC95%, IC: 51-58%), demonstrando que a adesão aos ADOs em pacientes com DM tipo 2 é subótima^(20)^.

Possível razão para o alto nível de adesão medicamentosa encontrada pode estar associado ao grupo ter sido caracterizado com a maioria de idosos. Revisão sistemática com metanálise sobre os fatores sociopessoais que afetam a adesão mostrou que os mais jovens possuem um risco 17% maior do que os mais velhos de não adesão ao tratamento^(21)^.

Do ponto de vista empírico, questões socioculturais e psicoespirituais são relacionadas à adesão ao tratamento. O apoio familiar foi um grande facilitador para adesão ao tratamento medicamentoso no DM tipo 2, ao passo que o custo dos medicamentos e a comunicação deficiente com os prescritores dos medicamentos foram barreiras^(22)^. No contexto psicoespiritual, as emoções positivas, como empoderamento e capacidade de seguir com o autocuidado diante dos benefícios dos tratamentos, e negativas, como medo, autoacusação, culpa, desamparo, frustação e preocupação com as complicações potenciais do diabetes negativas, influenciam a adesão ao tratamento medicamentoso no DM tipo 2^(23)^. Tais emoções possuem nexos com as perspectivas psicoespirituais. Evidências indicam que a inteligência espiritual e inteligência emocional podem se complementar para aumentar o autogerenciamento e os mecanismos adaptativos em pessoas com diabetes^(24)^, ou beneficiar o paciente por meio da espiritualidade e religiosidade, inclusive no enfrentamento de situações estressantes causadas pelas condições da doença^(25)^.

A relação entre conforto, outra variável identificada com bom nível, e adesão medicamentosa caracteriza a originalidade desta investigação. Não foram recuperadas na literatura investigações similares. Em paralelo, estudo desenvolvido na Turquia avaliou o nível de conforto de pessoas com DM tipo 2 utilizando QCG e a relação entre conforto e aceitação da doença^(9)^. Foi detectada uma correlação positiva e fraca entre a Escala de Aceitação da Doença e os escores médios do QCG e suas subdimensões. Na investigação^(9)^, não foram apresentadas as médias dos níveis de conforto, entretanto a escolaridade, a renda, a profissão, as pessoas com quem o paciente morava, a frequência de acompanhamento, a prática de dietas e exercícios físicos, e as complicações crônicas foram considerados preditores significativos para o nível de conforto geral.

Em paralelo aos preditores identificados^(9)^ e às variáveis investigadas nos participantes desta pesquisa, podemos hipotetizar razões pelas quais o nível de conforto detectado foi alto. Entre os preditores, destacamos o nível de escolaridade (baixa escolaridade identificada) e a renda (baixa renda identificada). A literatura compreende que o alto nível de escolaridade reduz os níveis de conforto, pois quanto mais informação, maior a chance de se preocupar com o prognóstico da doença e aumentar a carga psíquica^(9)^. Embora rendimentos mais elevados aumentem as expectativas em relação à doença e possam afetar negativamente o conforto, níveis de rendimento baixos não são suficientes para satisfazer essas expectativas^(9)^. Em contrapartida a esses preditores de conforto destacados, quando consideramos essas características com a adesão ao tratamento, o nível de escolaridade e a renda apresentam tanto relação positiva quanto negativa em estudos recuperados por revisões sistemáticas, característica da heterogeneidade das pesquisas realizadas nesta área^(21,26)^.

Estritamente pelas evidências desta investigação, não se suporta a afirmativa relacional de que a pessoa, ao alcançar o conforto por meio de alguma intervenção terapêutica, tem aumentada a probabilidade do envolvimento em comportamentos de procura de saúde^(7,8)^. Contudo, antes de desistir de qualquer interesse na proposição e na teoria como o todo, afirmamos que os achados são discutidos diante da afirmativa de “conhecimento geral é conhecimento teórico”. Por meio de sua abstração, teorias são aplicadas em locais e populações, mas achados empíricos, não^(19)^.

Pesquisadores, usualmente, avaliam a possibilidade de generalizar os achados obtidos naquele estudo, em particular para outras populações ou grupos de pessoas, outros tempos, locais, contextos ou outras categorias de situações singulares, contudo semelhantes, como um critério de utilidade de suas pesquisas^(19,27)^. Nas pesquisas desenhadas para o teste teórico, os alvos a serem testados podem ser conceitos abstratos, como são os conceitos de conforto e adesão. Desse modo, a validade externa deve ser considerada em função desse tipo mais abstrato de alvo^(28)^.

Se considerarmos adequados os instrumentos de medida dos conceitos de conforto e adesão (QCG e MAT), restariam problemas do desenho da pesquisa e/ou de deficiências da teoria em teste por meio de seus construtos. Em primeiro lugar, partimos do pressuposto de que existe uma limitação no desenho metodológico decorrente da falha em observar uma formulação da teoria que fundamentou a construção do QCG, apontando, portanto, para uma deficiência ilusória na plausibilidade da teoria.

O QCG, como um instrumento derivado de teoria, representa uma tentativa de trazer para o domínio empírico e operacional afirmações e demais elementos da Teoria do Conforto de Kolcaba. Embora o alcance dessa transferência da teoria para a mensuração possa sempre ser debatido, as escalas são aplicadas partindo da perspectiva de que aspectos da teoria estariam nelas incorporados. Diante disso, embora o QCG não comunique explicitamente, ele presume a existência de uma definição teórica para o conceito de conforto. Considerando os quatro contextos (físico, psicoespiritual, ambiental e sociocultural), o conforto seria um estado dinâmico, sujeito a rápidas mudanças positivas e negativas^(28)^.

Portanto, se, de fato, o estado do conforto for dinâmico e sujeito a rápidas mudanças, como a definição teórica comunica, então uma aplicação transversal do instrumento de medida não seria capaz de medir a correlação que deveria ser acompanhada por um desenho de método longitudinal. Assim, se, por um lado, no planejamento da pesquisa, não existisse contraindicação metodológica de aplicação do instrumento de forma transversal para investigar, por meio estatístico, a existência de correlação, por outro, a não observância da definição de conforto, uma afirmativa teórica não relacional, pode ser uma boa explicação por não se encontrar a correlação que foi estabelecida na hipótese, mesmo que ela possa existir.

Para um empirista mais radical, o limite do desenho metodológico associado à nulidade de correlação seria suficiente para encerrar o debate de qualquer possibilidade de relevância desta pesquisa. Entretanto, nem sempre a ciência avança em casos diretamente bem-sucedidos. Resultados identificados em outro grupo de investigação corroboram essa contradição. Pesquisa desenvolvida entre adolescentes com DM tipo 1 identificou, por meio da regressão logística, que o maior nível de conforto autorrelatado com as tarefas de controle do diabetes foi associado a uma maior hemoglobina glicada (p=0,006), após o controle de idade, sexo, raça, tipo de seguro saúde e duração do diabetes^(29)^. Destaca-se que o autorrelato do conforto com autogerenciamento independente do DM tipo 1 pode não ser uma métrica suficiente para avaliar a necessidade de mais intervenção para otimizar os resultados glicêmicos na fase de transição da adolescência para idade adulta^(29)^. Não existem aparentes restrições metodológicas na mencionada pesquisa^(29)^. Entretanto, por mais uma vez, a teoria informa aspectos para construção do conhecimento geral, modulando as questões de validade externa.

Quando uma proposição teórica de uma relação esperada entre dois conceitos não é verificada por estudo na condição natural, duas considerações podem se apresentar *a priori:* o teste possui baixa validade externa, ou se o teste for bem desenhado, existem entre os conceitos fatores que a teoria não especifica. Assim, explicitamente, “se os testes experimentais podem manipular todas as variáveis teoricamente significativas e nada mais, então um problema com a validade externa de um experimento é um problema com a utilidade da teoria”^(19)^.

### Limitações do estudo

Este estudo observacional foi diretamente impactado pela dificuldade de localizar as afirmativas teóricas críticas para o teste teórico, isso porque a definição teórica empregada para o conceito de conforto na construção do QCG e da sua adaptação cultural para o Brasil omitia esta propriedade de dinamicidade temporal^(17)^, sendo a consideração como estado dinâmico, constando apenas de uma publicação dirigida a situações de perianestesia^(28)^. Desse modo, o desenho metodológico transversal desconsiderou a especificidade temporal do conceito a ser medido, apontando para uma limitação da aplicação isolada do QCG, especialmente em estudos de teste teórico.

Destaca-se, ainda, a formação da amostra analisada pela seleção não probabilística. Além disso, o estudo considerou o ambiente de atendimento em saúde, desconsiderando o ambiente em que os participantes realizam o tratamento medicamentoso, seja ele domiciliar ou local de trabalho. Outro ponto de limitação foi a impossibilidade de verificação associada da adesão aos que fazem uso de insulina e ADOs, uma vez que o instrumento era separado, impossibilitando análise conjunta dos indivíduos que utilizam ambos os regimes medicamentosos, optando-se pela análise apenas da insulina nesses casos.

### Contribuições para a área da enfermagem

Os resultados desta pesquisa ampliam o corpo de conhecimento da enfermagem ao investigar, de forma inédita no contexto nacional brasileiro, a relação entre o construto conforto e a adesão medicamentosa de pessoas com DM tipo 2. Ainda que não tenha sido observada correlação estatisticamente significativa entre as variáveis, os achados oferecem subsídios importantes para a prática, ao evidenciar os níveis de conforto percebidos pelos participantes e o padrão de adesão ao uso de ADOs ou insulina.

Esses achados contribuem para reflexão crítica sobre os fatores que influenciam a adesão ao tratamento, reforçando a necessidade de abordagens multifatoriais que incluam, além do conforto, aspectos como suporte social, educação em saúde, motivação, e manejo de barreiras individuais e coletivas. Do ponto de vista teórico, os resultados fornecem elementos para o refinamento da aplicação da Teoria do Conforto de Kolcaba em estudos voltados a comportamentos de saúde, incentivando novas pesquisas que explorem interações com outras variáveis clínicas, psicossociais, culturais e sua temporalidade.

No âmbito assistencial, o estudo aponta que o planejamento do cuidado de enfermagem deve considerar o conforto como componente essencial para o bem-estar e qualidade de vida, mas que sua influência isolada sobre a adesão pode ser limitada, demandando intervenções integradas e personalizadas.

## CONCLUSÕES

Embora tenham se observado bom estado de conforto geral e boa adesão medicamentosa nos participantes da pesquisa, a hipotética correlação entre o conforto da teoria de Kolcaba e o construto adesão medicamentosa parece ter sido refutada, visto que se verificou correlação nula entre as variáveis. No entanto, nossa discussão teórica compreendeu que a definição do construto conforto, proveniente da teoria de Kolcaba, retrata um estado dinâmico e sujeito a rápidas mudanças. Isto pode explicar a não correlação entre os construtos em investigação transversal, o que não refuta a hipotética existência de correlação em uma perspectiva longitudinal. Dessa forma, estudos futuros podem avançar o conhecimento para elucidar a hipótese teórica.

## Data Availability

Os dados de pesquisa estão disponíveis no corpo do artigo.
